# Association Between XPD Lys751Gln and Asp312Asn Polymorphisms and Hepatocellular Carcinoma Risk: A Systematic Review And Meta-Analysis

**DOI:** 10.1097/MD.0000000000000330

**Published:** 2014-12-02

**Authors:** Qiliu Peng, Shan Li, Xianjun Lao, Zhiping Chen, Ruolin Li, Xue Qin

**Affiliations:** From the Department of Clinical Laboratory (QP, SL, XL, XQ), First Affiliated Hospital of Guangxi Medical University; Department of Occupational Health and Environmental Health (ZC), School of Public Health, Guangxi Medical University; and Department of Medicine Research (RL), First Affiliated Hospital of Guangxi Medical University, Nanning, Guangxi, China.

## Abstract

Genetic polymorphisms of xeroderma pigmentosum group D (XPD) in the nucleotide excision repair pathway may influence cancer susceptibility by affecting the capacity for DNA repair. Studies investigating the association between XPD Lys751Gln and Asp312Asn polymorphisms and hepatocellular carcinoma (HCC) risk reported inconsistent results. The aim of this study was to quantitatively summarize the evidence for such an association.

Eligible studies were identified by searching electronic databases including PubMed, Embase, Cochrane library, and CBM, Chinese Biomedical Literature Database, for the period up to October 2014. The association of XPD Lys751Gln and Asp312Asn polymorphisms and HCC risk was assessed by odds ratios (ORs) together with their 95% confidence intervals (CIs).

Finally, a total of 11 studies with 4322 cases and 4970 controls were included for XPD Lys751Gln polymorphism and 6 studies with 2223 cases and 2441 controls were available for XPD Asp312Asn polymorphism. With respect to XPD Lys751Gln polymorphism, statistically significant increased HCC risk was found when all studies were pooled into the meta-analysis (Gln/Gln vs Lys/Lys: OR = 1.363, 95% CI 1.065–1.744, *P* = 0.014; Lys/Gln vs Lys/Lys: OR = 1.205, 95% CI 1.099–1.321, *P* = 0.000; Gln/Gln+Lys/Gln vs Lys/Lys: OR = 1.300, 95% CI 1.141–1.480, *P* = 0.000). In subgroup analyses by ethnicity, source of control, Hardy–Weinberg equilibrium (HWE) in controls, hepatitis B virus (HBV) infection, and statistically significant increase of HCC risk was found in East Asians, population-based studies, studies consistent with HWE, and HBV-positive subjects, but not in mixed/other populations, hospital-based studies, studies deviating from HWE, and HBV-negative subjects. With respect to XPD Asp312Asn polymorphism, no significant association with HCC risk was found in the overall and subgroup analyses.

The results suggest that the XPD Lys751Gln polymorphism contributes to increased HCC susceptibility, especially in East Asian populations. Further, large and well-designed studies are required to validate this association.

## INTRODUCTION

Hepatocellular carcinoma (HCC) is the fifth most common cancer and the third common cause of cancer mortality worldwide.^1^ The distribution of HCC is imbalanced throughout the world, with the highest incidence rates in East and Southeast Asia and Sub-Saharan Africa, and China alone accounted for >50% of all HCC malignancies.^1–2^ Etiologically, carcinogenesis of HCC is a complex, multistep, and multifactor process, in which many factors are implicated.^3^ As we know, chronic infection with hepatitis B virus (HBV) or hepatitis C virus (HCV), and obesity-related nonalcoholic steatohepatitis (NASH) are the most well-established environmental risk factors for HCC worldwide.^4,5^ However, only a fraction of lifelong HBsAg carriers and NASH subjects eventually develop HCC, and only 2.5% of HCV-infected individuals develop HCC later in life.^6^ The exact mechanism of hepatocarcinogenesis is still incompletely understood, and the risk factors for HCC still need to be further elucidated.

Human cancer can be initiated by DNA damage caused by endogenous and exogenous mutagens such as ionizing radiation, UV, viruses, and environmental chemical agents.^3^ Importantly, to counteract the deleterious consequences of DNA-damaging agents, humans have developed a set of complex DNA repair systems that as a whole take care of most of the insults inflicted on a cell's vital genetic information. The DNA repair systems play critical roles in maintaining the functions of normal cells and genomic integrity through the reversal of the damaged DNA.^7^ Among the DNA repair systems, the nucleotide excision repair (NER) pathway constitutes the primary mechanism for removal of bulky adducts from DNA, and thus is an important part of the cellular defense against a large variety of structural unrelated DNA lesions generated by ionizing radiation and strong alkylating agents as well as lesions formed by endogenous DNA-damaging agents like viruses.^8^

Xeroderma pigmentosum group D (XPD), also named excision repair cross-complimentary group 2, is 1/8 core genes (ie, ERCC1, XPA, XPB, XPC, XPD, XPE, XPF, and XPG) in the NER pathway of the DNA repair system. XPD maps to chromosome 19q13.3 and is composed of 23 exons.^9^ It codes for a DNA helicase subunit of the core transcription factor IIH, which is essential for NER and transcription and plays a critical role in transcription-coupled NER pathway.^10^ Single nucleotide polymorphisms (SNPs) in the exons of XPD may influence the protein activity, resulting in defects in the NER pathway and reduced DNA repair capacity. The Lys751Gln and Asp312Asn were the 2 most commonly studied SNPs in the coding region of the XPD gene.^11^ The XPD Lys751Gln polymorphism (rs13181) is characterized by an A to C substitution resulting in a lysine (Lys) to glutamine (Gln) amino acid exchange at position 751 in exon 23, whereas the Asp312Asn polymorphism (rs1799793) is characterized by a G to A transition causing an aspartic acid (Asp) to asparagine amino acid (Asn) exchange at position 312 in exon 10. Previous studies have demonstrated that the 2 polymorphisms were associated with lower DNA repair capacity and higher level of DNA adducts.^12–14^ Hence, it is biologically reasonable to hypothesize a potential relationship between the XPD Lys751Gln and Asp312Asn polymorphisms and HCC risk.

Over the last 2 decades, a number of epidemiological studies have been conducted to investigate the associations between XPD Lys751Gln and Asp312Asn polymorphisms and HCC risk, but the results remain inconsistent and underpowered. With respect to XPD Lys751Gln polymorphism, a meta-analysis by Zhang and Mou^15^ did not find any evidence of significant association between XPD Lys751Gln polymorphism and HCC risk in the overall populations and subgroup analysis. However, the evidence was limited because they failed to include all eligible studies in the meta-analysis.^16–20^ In addition, the source of heterogeneity was not extensively explored in this study. With respect to XPD Asp312Asn polymorphism, to the best of our knowledge, no meta-analyses on this issue have ever appeared. The exact relationship between genetic polymorphisms of XPD Lys751Gln and Asp312Asn and HCC susceptibility has not been entirely established. To provide the most comprehensive assessment of the associations between the XPD Lys751Gln and Asp312Asn polymorphisms and HCC risk, we performed an updated meta-analysis of all available studies.

## MATERIAL AND METHODS

### Literature Search

We conducted a comprehensive literature search in PubMed, EMBASE, Cochrane library, and CBM, Chinese Biomedical Literature Database, up to October 01, 2014 using the following search strategy: (“hepatocellular carcinoma,” “HCC,” or “liver cancer”) and (“Xeroderma pigmentosum complementation group D,” “XPD,” “excision repair cross-complimentary group 2” or “ERCC2”). There was no restriction on sample size, language, population, or type of report. All eligible studies were retrieved and their references were checked for other relevant studies. In addition, we also used the “Related Articles” function in PubMed to search for other potential relevant articles. When several studies reported on the same or overlapping data, we chose the most recent or largest population. The meta-analysis was performed according to the proposal of Meta-Analysis of Observational Studies in Epidemiology group.^21^

### Selection Criteria

We reviewed titles and abstracts of all citations and retrieved studies. The following inclusion criteria were used for literature selection: case–control studies that evaluated the association between XPD Lys751Gln and Asp312Asn polymorphisms and HCC risk; used an unrelated case–control design; presented an odds ratio (OR) together with 95% confidence interval (CI) or other information for estimating OR (95% CI); and the control group did not contain malignant tumor patients. Conference abstracts, review articles, meta-analyses, case reports, editorials, and letters were excluded.

### Data Extraction

Data were extracted from each study by 2 authors independently (X.L. and Q.P.) according to the selection criteria listed above. Decisions were compared and disagreements were resolved by consensus or by involving a third author (X.Q.). The following information was extracted from each study: first author, publication year, country of origin, ethnicity, genotyping method, source of control, matching variables, HCC diagnosis, total number of cases and controls, and genotype frequencies of cases and controls. When the genotype frequency or other important information was not reported, we contacted the author to get the relevant information by e-mail or telephone.

### Statistical Analysis

The strength of the association between XPD Lys751Gln and Asp312Asn polymorphisms and HCC risk was assessed by ORs together with 95% CIs. The significance of the pooled ORs was determined by *Z* test, and the *P* values <0.05 were considered significant. We evaluated the XPD Lys751Gln and Asp312Asn polymorphisms and HCC risk using codominant, recessive, and dominant models.

The χ^2^-based *Q* test was used to assess the statistical heterogeneity among studies.^22^ If the result of the *Q* test was *P*_*Q*_* *<* *0.10, suggesting the existence of heterogeneity, the pooled ORs were calculated using the random-effects model (the DerSimonian and Laird method).^23^ Otherwise, when the result of the *Q* test was *P*_*Q*_ ≥ 0.1, indicating the absence of heterogeneity, the fixed-effects model (the Mantel–Haenszel method)^24^ was applied. Logistic metaregression and subgroup analyses were used to identify the sources of heterogeneity among studies. The following parameters were included as covariates in metaregression analysis: ethnicity (East Asians vs mixed/other), source of controls (hospital-based vs population-based studies), genotyping methods (polymerase chain reaction-restriction fragment length polymorphism vs no polymerase chain reaction-restriction fragment length polymorphism), and Hardy–Weinberg equilibrium (HWE) in controls (Yes vs No). Subgroup analyses were performed according to ethnicity, source of control, HWE status, and HBV infection. Galbraith plot analysis was performed to further explore the source of heterogeneity.

Sensitivity analysis was performed by sequential exclusion of the individual studies to assess the robustness of the results. Begg funnel plot and Egger regression asymmetry test were performed to evaluate the publication bias. If the publication bias presented, the Duval and Tweedie^25^ nonparametric “trim and fill” method was applied to adjust for it. The HWE was evaluated by the goodness-of-fit χ^2^ test for the control groups of each study (significance set at *P* = 0.05, *P* < 0.05 was considered a departure from HWE). All analyses were performed using Stata software, version 12.0 (Stata Corp, College Station, TX). All *P* values were 2-sided. To ensure the reliability and the accuracy of the results, 2 authors entered the data into the statistical software programs independently with the same results.

## RESULTS

### Study Characteristics

Based on our search strategy, 34 records were found, but only 14 full-text studies were preliminarily identified for further detailed evaluation after screening the titles and abstracts. According to the exclusion criteria, 3 studies were excluded including 1 presenting insufficient data for calculating OR and 95% CI,^26^ 1 was a review,^27^ and 1 was a meta-analysis.^15^ Manual search of references cited in the retrieval studies identified 1 additional study.^20^ As a result, a total of 12 relevant studies (1 was a dissertation by a postgraduate student) met the inclusion criteria for the meta-analysis.^16–20,28–34^ The main characteristics of the studies were presented in Table 1. Among them, 11 studies with 4322 cases and 4970 controls were included for XPD Lys751Gln polymorphism and 6 studies with 2223 cases and 2441 controls were available for XPD Asp312Asn polymorphism. The sample size in these studies varied considerably, ranging from 124 to 3482 individuals. Of all the eligible studies, 10 were conducted on East Asians and 1 was on mixed/other populations for the XPD Lys751Gln polymorphism; 6/6 studies were conducted on East Asians for the XPD Asp312Asn polymorphism. One study in the present meta-analysis did not provide definite criteria for HCC diagnosis.^33^ The genotype distributions of the controls in 2 studies were inconsistent with HWE for the XPD Lys751Gln polymorphism^17,31^ and 2 were inconsistent with HWE for the XPD Asp312Asn polymorphism.^16,18^

**TABLE 1:**
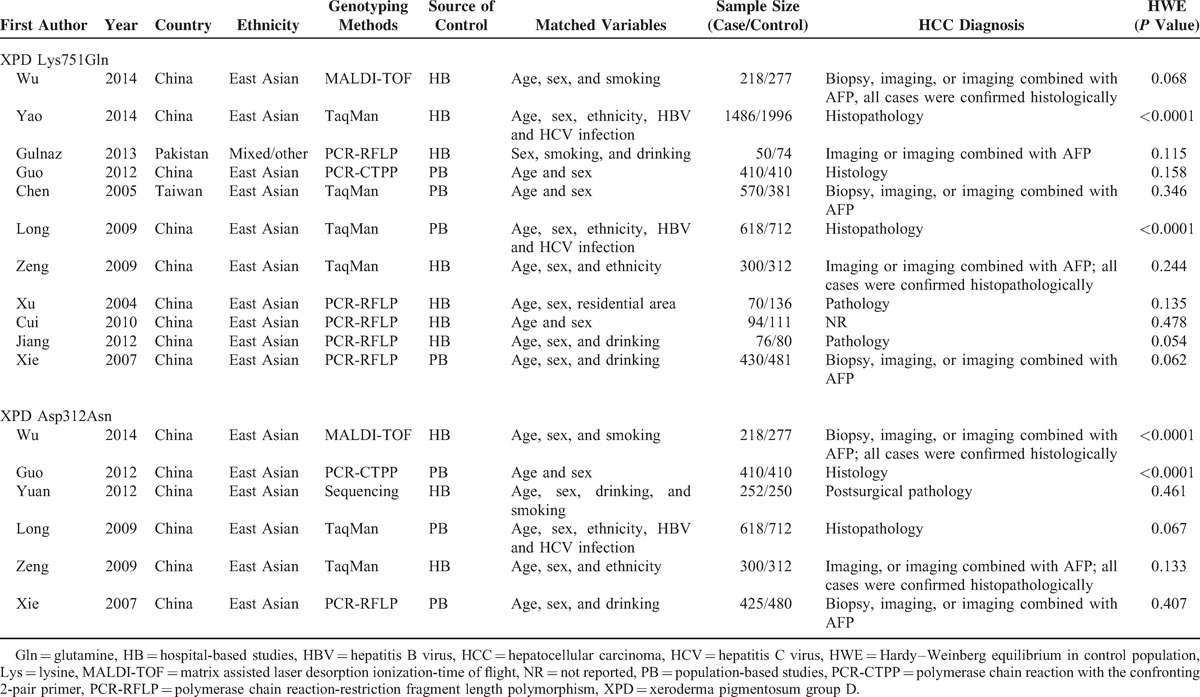
Characteristics of the Included Studies

### Meta-Analysis Results

**FIGURE 1:**
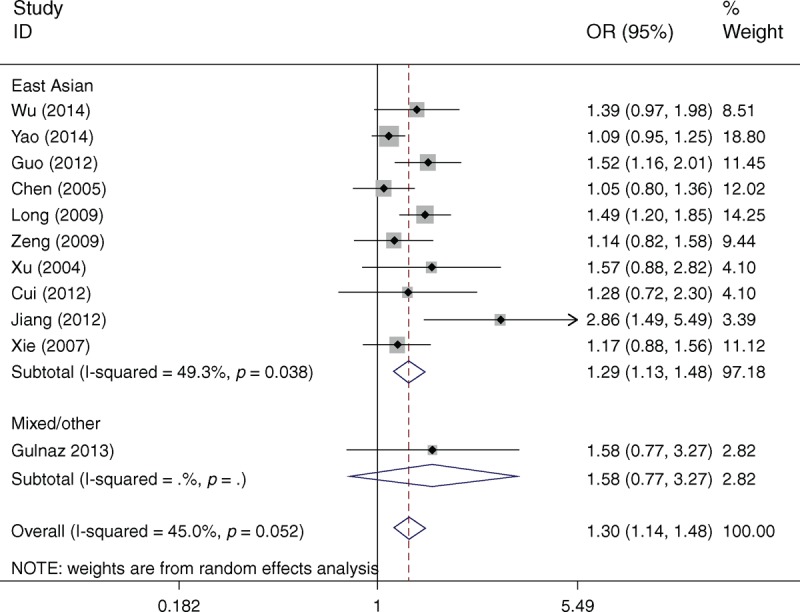
Forest plots of XPD Lys751Gln polymorphism and HCC risk in subgroup analysis by ethnicity using a random-effects model (dominant model Gln/Gln+Lys/Gln vs Lys/Lys). Gln = glutamine, HCC = hepatocellular carcinoma, Lys = lysine, OR = odds ratio, XPD = xeroderma pigmentosum group D.

Table 2 lists the main results of meta-analysis of XPD Lys751Gln polymorphism and HCC risk. Overall, statistically significant increased HCC risk was found when all studies were pooled into the meta-analysis (Gln/Gln vs Lys/Lys: OR = 1.363, 95% CI 1.065–1.744, *P* = 0.014; Lys/Gln vs Lys/Lys: OR = 1.205, 95% CI 1.099–1.321, *P* = 0.000; Gln/Gln+Lys/Gln vs Lys/Lys: OR = 1.300, 95% CI 1.141–1.480, *P* = 0.000). In subgroup analysis by ethnicity, statistically significant increased HCC risk was found in East Asians (Gln/Gln vs Lys/Lys: OR = 1.372, 95% CI 1.058–1.777, *P* = 0.017; Lys/Gln vs Lys/Lys: OR = 1.199, 95% CI 1.093–1.316, *P* = 0.000; Gln/Gln+Lys/Gln vs Lys/Lys: OR = 1.294, 95% CI 1.131–1.481, *P* = 0.000; Figure 1) but not in mixed/other populations. In subgroup analysis according to source of control, significantly increased HCC risk was found in population-based studies (Lys/Gln vs Lys/Lys: OR = 1.353, 95% CI 1.181–1.551, *P* = 0.000; Gln/Gln+Lys/Gln vs Lys/Lys: OR = 1.323, 95% CI 1.168–1.498, *P* = 0.000), but not in hospital-based studies. When stratified by HWE in controls, significantly increased HCC risk was observed in studies consistent with HWE (Lys/Gln vs Lys/Lys: OR = 1.328, 95% CI 1.164–1.516, *P* = 0.000; Gln/Gln+Lys/Gln vs Lys/Lys: OR = 1.295, 95% CI 1.147–1.463, *P* = 0.000) but not in studies inconsistent with HWE. In subgroup analysis according to HBV infection, significantly increased HCC risk was identified in HBV-positive patients (Lys/Gln vs Lys/Lys: OR = 1.502, 95% CI 1.094–2.157, *P* = 0.007; Gln/Gln+Lys/Gln vs Lys/Lys: OR = 1.431, 95% CI 1.046–2.099, *P* = 0.013) but not in HBV-negative subjects.

**TABLE 2:**
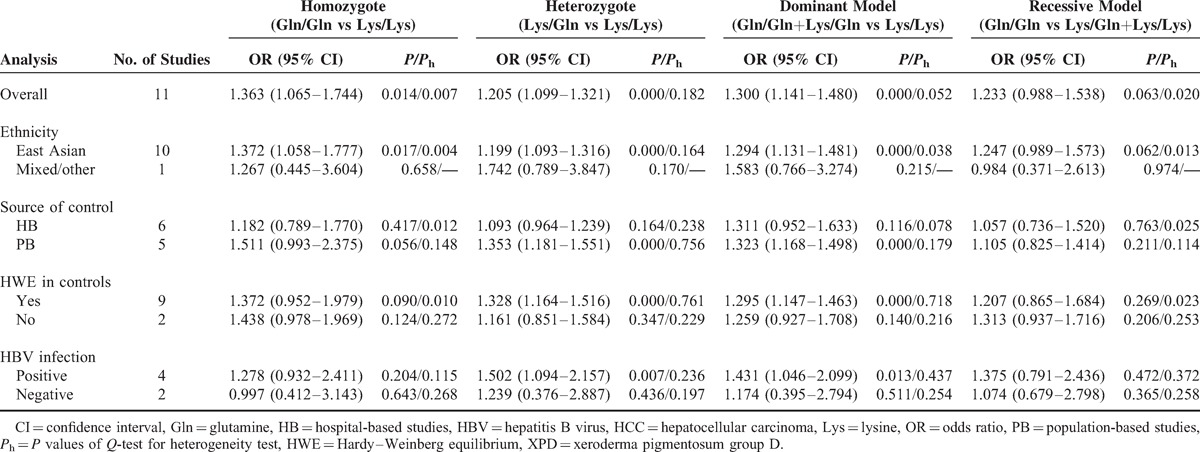
Meta-Analysis of XPD Lys751Gln Polymorphism and HCC Risk

**FIGURE 2:**
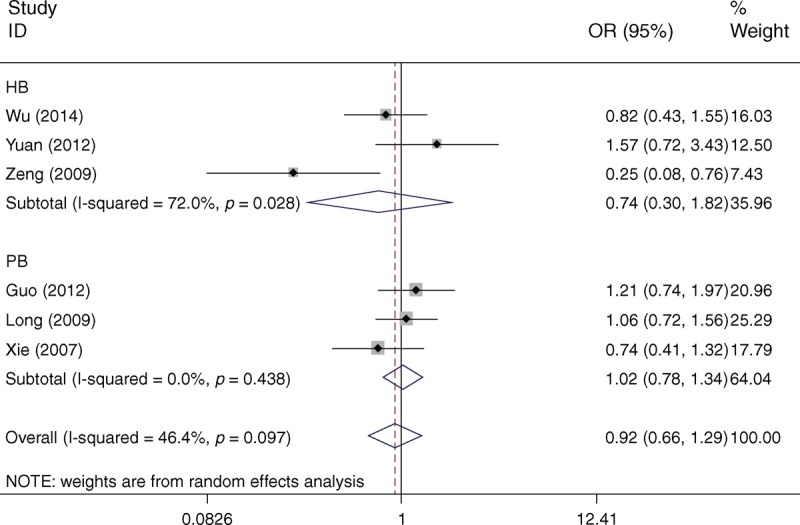
Forest plots of XPD Asp312Asn polymorphism and HCC risk in subgroup analysis by source of controls using a random-effects model (recessive model Asn/Asn vs Asp/Asn+Asp/Asp). Asn = asparagine amino acid, Asp = aspartic acid, HB = hospital-based studies, HCC = hepatocellular carcinoma, OR = odds ratio, PB = population-based studies, XPD = xeroderma pigmentosum group D.

Table 3 lists the main results of meta-analysis of XPD Asp312Asn polymorphism and HCC risk. There was no evidence of significant association between the XPD Asp312Asn polymorphism and HCC risk when all eligible studies were pooled into the meta-analysis (Asn/Asn vs Asp/Asp: OR = 0.959, 95% CI 0.761–1.208, *P* = 0.720; Asp/Asn vs Asp/Asp: OR = 1.048, 95% CI 0.920–1.195, *P* = 0.479; Asn/Asn+Asp/Asn vs Asp/Asp: OR = 1.031, 95% CI 0.913–1.163, *P* = 0.625; Asn/Asn vs Asp/Asn+Asp/Asp: OR = 0.925, 95% CI 0.660–1.295, *P* = 0.648). In subgroup analyses by ethnicity, source of controls, HWE in controls, and HBV infection, statistically significant association was also not detected in all subgroups (Figure 2).

**TABLE 3:**
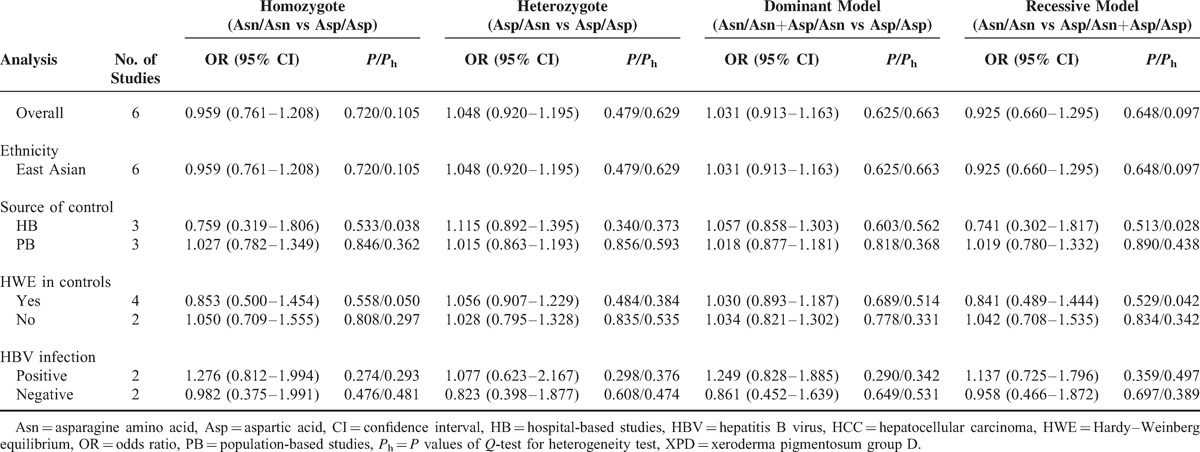
Meta-Analysis of XPD Asp312Asn Polymorphism and HCC Risk

### Heterogeneity Analysis

**FIGURE 3:**
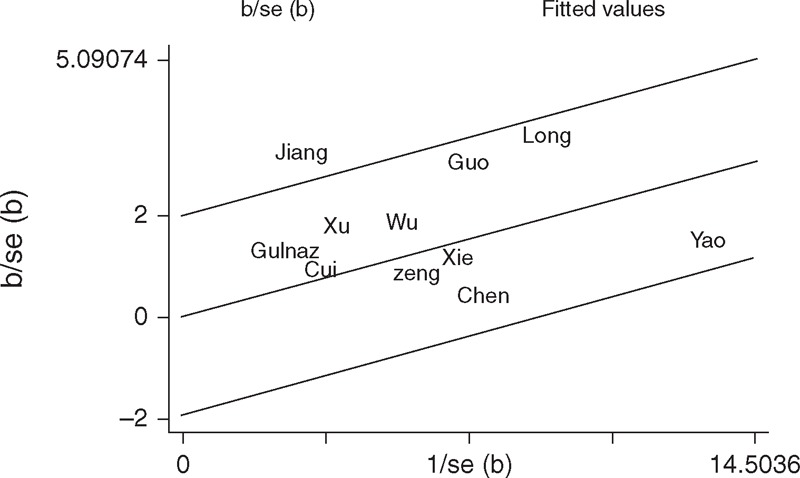
Galbraith plots of XPD Lys751Gln polymorphism and HCC risk (dominant model Gln/Gln+Lys/Gln vs Lys/Lys). The study by Jiang et al^20^ was spotted as the outlier. Gln = glutamine, HCC = hepatocellular carcinoma, Lys = lysine, XPD = xeroderma pigmentosum group D.

With respect to XPD Lys751Gln polymorphism, statistically significant between-study heterogeneity was found in the pooled analyses of total eligible studies (Gln/Gln vs Lys/Lys: *P*_h_ = 0.007; Gln/Gln+Lys/Gln vs Lys/Lys: *P*_h_ = 0.052; Gln/Gln vs Lys/Gln+Lys/Lys: *P*_h_ = 0.020; Table 2). To investigate the sources of heterogeneity, we performed metaregression and subgroup analyses. Metaregression revealed that the ethnicity, source of controls, genotyping methods, and HWE in controls were not effect modifiers. Subsequently, we performed subgroup analyses by ethnicity, source of control, HWE in controls, and HBV infection. However, heterogeneity still existed in East Asians, hospital-based studies, and studies consistent with HWE (Table 2). To further explore the source of heterogeneity, we performed Galbraith plot analysis to identify the outlier that might contribute to the heterogeneity. The results indicated that the study by Jiang et al^20^ was the outlier in the overall populations (Figure 3). All *P*_h_ values in the overall populations, East Asians, hospital-based studies, and studies consistent with HWE were >0.10 after excluding the study by Jiang et al.^20^ Interestingly, the significance of the pooled ORs for the XPD Lys751Gln polymorphism and HCC risk in the overall populations, East Asians, hospital-based studies, and studies consistent with HWE did not change after omitting this study.

**FIGURE 4:**
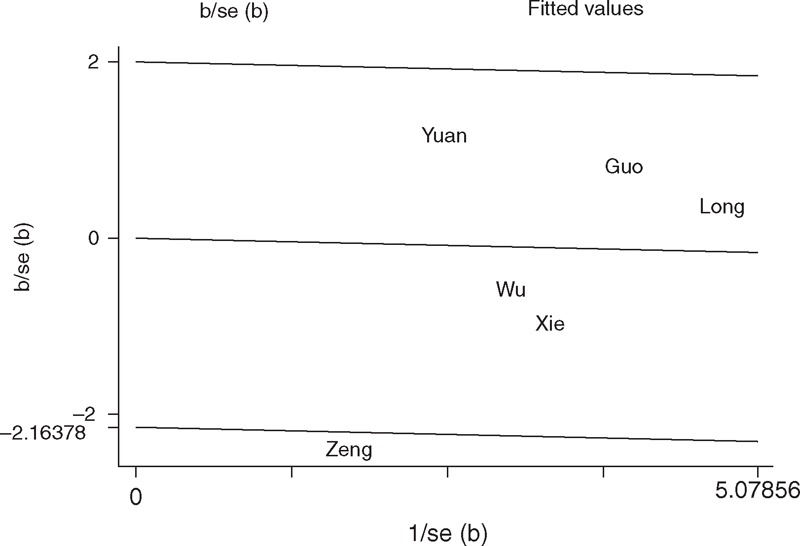
Galbraith plots of XPD Asp312Asn polymorphism and HCC risk (recessive model Asn/Asn vs Asp/Asn+Asp/Asp). The study by Zeng et al^19^ was spotted as the outlier. Asn = asparagine amino acid, Asp = aspartic acid, HCC = hepatocellular carcinoma, XPD = xeroderma pigmentosum group D.

With respect to XPD Asp312Asn polymorphism, the *P*_h_ value of the *Q* test was <0.10 in the recessive model in the overall populations (Asn/Asn vs Asp/Asn+Asp/Asp: *P*_h_ = 0.097; Table 3), indicating statistically significant heterogeneity among studies. Metaregression analysis revealed that the ethnicity, source of control, genotyping methods, and HWE in controls were not effect modifiers. Subgroup analyses indicated that the heterogeneity was evident in East Asians, hospital-based studies, and studies consistent with HWE. Galbraith plot analysis suggested that the study by Zeng et al^19^ was the outlier and the major source of the heterogeneity (Figure 4). The *P*_h_ values were >0.10 after excluding the study by Zeng et al^19^ in the overall populations, East Asians, hospital-based studies, and studies consistent with HWE. However, the significance of the pooled ORs for the XPD Asp312Asn polymorphism and HCC risk in the overall populations, East Asians, hospital-based studies, and studies consistent with HWE were also not changed by excluding this study.

### Sensitivity Analysis

Sensitivity analysis was performed by sequential omission of individual studies for both the XPD Lys751Gln and Asp312Asn polymorphisms. For analyses of pooling >3 studies, the significance of ORs was not materially influenced by omitting any single study. For the XPD Lys751Gln polymorphism, sensitivity analysis was further performed by excluding the studies by Yao et al^17^ and Long et al^31^ in which the control populations were significantly deviated from HWE. The significance of all ORs was not altered after excluding the 2 studies. For the XPD Asp312Asn polymorphism, sensitivity analysis was also performed by excluding those 2 studies by Wu et al^16^ and Guo et al^18^ in which the control populations were inconsistent with HWE, and the significance of all ORs was also not altered.

### Publication Bias

**FIGURE 5:**
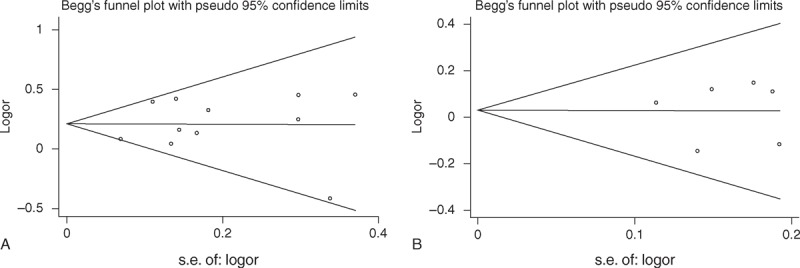
Funnel plot analysis to detect the publication bias. Each point represents a single study for the indicated association. (A) Funnel plot for XPD Lys751Gln polymorphism in overall analysis (dominant model Gln/Gln+Lys/Gln vs Lys/Lys: *P* = 0.543). (B) Funnel plot for XPD Asp312Asn polymorphism in overall analysis (recessive model Asn/Asn vs Asp/Asn+Asp/Asp: *P* = 0.977). Asn = asparagine amino acid, Asp = aspartic acid, Gln = glutamine, Lys = lysine, XPD = xeroderma pigmentosum group D.

Begg funnel plot and Egger test were used to access the publication bias of literatures. The shape of funnel plots did not reveal any evidence of obvious asymmetry (Figure 5), and the *P* values of the Egger tests for both the XPD Lys751Gln and Asp312Asn polymorphisms were all >0.05, providing statistical evidence of the funnel plots’ symmetry. The results suggested that publication bias did not exist in this study.

## DISCUSSION

NER pathway of the DNA repair systems is the primary mechanism for the removal of bulky adducts from DNA, and thus is an important part of the cellular defense against a large variety of structural unrelated DNA lesions.^35,36^ XPD is one of the most important proteins in NER and closely associated with NER pathway coordination by interacting with most components of the NER short-patch pathway. Genetic polymorphisms and mutations in XPD may influence the protein activity, resulting in defects in the NER pathway and reduced DNA repair capacity^37^; thus, modulating cancer susceptibility including HCC. To date, emerging epidemiological studies have evaluated the association of XPD Lys751Gln and Asp312Asn polymorphisms with HCC risk, but the results remain controversial and underpowered. Therefore, we performed this meta-analysis including all available studies to provide the most comprehensive assessment of the associations. The results based on 12 studies suggested that the XPD Lys751Gln polymorphism was significantly associated with increased HCC risk. However, our data did not support a genetic association of the XPD Asp312Asn polymorphism with HCC susceptibility.

XPD is an important DNA repair gene in the NER pathway. XPD Lys751Gln polymorphism is located at position 751 in exon 23 of the XPD gene with an A to C substitution leading to a Lys to Gln amino acid exchange. Inherited functional polymorphisms in exons of the XPD gene may influence the protein function, resulting in differences of the individual NER and DNA repair capacity that may affect the susceptibility to cancers. It has been reported that the Gln allele of the Lys751Gln polymorphism within the XPD gene was associated with higher DNA adduct levels and lower DNA repair efficiency.^35,38^ Therefore, it is biologically plausible that the XPD Lys751Gln polymorphism plays a critical role in hepatocarcinogenesis. This hypothesis was confirmed by our meta-analysis.

In the present study, we observed that the XPD Lys751Gln polymorphism presented a risk factor for HCC in East Asians, but not in mixed/other populations. When we excluded the study by Zhang and Mou,^15^ which was shown as an outlier in Galbraith plot analysis, statistically significant increased HCC risk was still found in the overall populations and East Asians but not in the mixed/other populations. The inconsistent results among diverse ethnicities demonstrated different effects of the XPD Lys751Gln polymorphism on HCC risk in different ethnic genetic backgrounds. Nevertheless, because of the small number of eligible studies among mixed/other populations, the observed association between the XPD Lys751Gln polymorphism and HCC risk in mixed/other populations is likely to be caused by chance because a study with small sample sizes may have insufficient statistical power to detect a slight effect or may have generated a fluctuated estimation. In this study, there was only 1 study for mixed/other populations concerning the XPD Lys751Gln polymorphism on HCC risk.^39^ Therefore, the results from the mixed/other populations should be interpreted with caution.

It was reported that hospital-based studies have inherent selection bias because of the fact that the controls just represent a sample of ill-defined reference population and may not be representative of the study population or the general population, particularly when the genotypes under investigation were associated with the disease-related conditions that the hospital-based controls may have.^40^ Selecting of proper and representative population-based controls is important in eliminating biases in such genetic association studies. Therefore, we performed subgroup analysis stratified by source of controls. The results revealed that the XPD Lys751Gln polymorphism was associated with an increased HCC risk in population-based studies, but not in hospital-based studies. Therefore, a methodologically preferable design, such as using a proper and representative population-based high quality study, is of great importance in case–control studies.

Evidence has demonstrated that studies disobeyed the law of HWE may be as a result of genetic reasons including nonrandom mating, or the alleles reflect recent mutations that have not reached equilibrium, as well as methodological reasons including biased selection of subjects from the population or genotyping errors.^41^ Because of reasons of disequilibrium, the results of genetic association studies might be unreliable when the distributions of genotypes in the control groups deviate from HWE. In the present study, the genotype distributions of the controls in 2 studies were inconsistent with HWE for the XPD Lys751Gln polymorphism.^17,31^ Therefore, we performed subgroup analysis according to HWE in controls. When excluding the studies that were inconsistent with HWE, the results were persistent and robust, suggesting that this factor probably had little effect on the overall estimates.

Hepatocarcinogenesis has been found to be closely correlated with chronic HBV or HCV infection, and in fact, a certain proportion of chronically infected HBV and HCV individuals will develop HCC. HBV belongs to a family of DNA viruses called hepadnaviruses. The oncogenic potential of HBV has been attributed to its ability to integrate into host cellular DNA, which impairs the genomic integrity and activates neighboring cellular genes directly to offer a selective growth advantage to liver cells. In addition, the production of hepatitis B x protein can serve as a transactivator on various cellular genes for cell growth and tumorigenesis. Therefore, there might be differences in carcinogenetic mechanisms of HCC between the HBV-positive patients and HBV-negative subjects. In the present study, we found that the XPD Lys751Gln polymorphism was significantly associated with increased HCC risk in HBV-positive patients but not in HBV-negative subgroup, which was consistent with the studies conducted by Long et al^31^ and Xu et al.^32^ The possible mechanism for the preferentially increased HCC risk of XPD Lys751Gln polymorphism in HBV-positive patients is that the DNA repair functions of the XPD have been reduced by the XPD Lys751Gln polymorphism, making them more vulnerable to cancer development. However, the hypotheses need to be proved in future studies.

The major concern in a sound meta-analysis is the degree of heterogeneity that exists between studies because heterogeneous data are prone to produce misleading results, and finding the sources of heterogeneity is one of the most important goals in a meta-analysis.^42^ In this study, statistically significant between-study heterogeneity was observed in the pooled analyses of total eligible studies. To identify the sources of heterogeneity, we performed subgroup analyses and metaregression. Subgroup analyses indicated that the heterogeneity was still evident in most of the subgroups for both the XPD Lys751Gln and Asp312Asn polymorphisms. Metaregression analysis revealed that none of the investigated variables was the source of heterogeneity for the 2 SNPs. Subsequently, we performed Galbraith plots to further investigate the heterogeneity. For the XPD Lys751Gln polymorphism, Galbraith plots spotted 1 study^20^ as the outlier and the possible source of heterogeneity. For the XPD Asp312Asn polymorphism, Galbraith plots also spotted 1 study^19^ as the outlier and the possible source of heterogeneity. When excluding the study by Jiang et al^20^ for the XPD Lys751Gln polymorphism and the study by Zeng et al^19^ for the XPD Asp312Asn polymorphism, all *P*_h_ values in the overall populations and subgroup analyses were >0.10. Interestingly, the summary ORs for the XPD Lys751Gln and Asp312Asn polymorphisms in different comparison models in the overall population and subgroup analyses were not materially changed by excluding the outlier studies, suggesting that our results were robust and reliable.

Despite our efforts to perform a comprehensive analysis, limitations in this study should be acknowledged. First, in subgroup analysis by ethnicity, the included studies regarded only East Asians and mixed/other populations. Data concerning other ethnicities such as whites and Africans were not found. Therefore, additional studies are needed to evaluate the effect of this functional polymorphism on HCC risk in different ethnicities. Second, the controls in the eligible studies were not uniformly defined. Although the controls were mainly selected from healthy populations, some had benign diseases such as liver cirrhosis, HBsAg positivity, and so on. Therefore, nondifferential misclassification bias was possible because these studies may have included the control populations who have different risks of developing HCC. Third, our results were based on unadjusted estimates. We did not perform analysis adjusted for other covariates such as sex, age, drinking and smoking status, HBV and HCV carrier status, environmental factors, and so on because of the unavailable original data in the eligible studies.

Despite the limitations, the results suggest that the XPD Lys751Gln polymorphism contributes to increased HCC susceptibility, especially in East Asians. Further, large and well-designed studies are required to validate this association.

## References

[1] Jemal A Bray F Center MM Global cancer statistics. *CA Cancer J Clin* 2011; 61:69–90. 21296855 10.3322/caac.20107

[2] Raza SA Clifford GM Franceschi S Worldwide variation in the relative importance of hepatitis B and hepatitis C viruses in hepatocellular carcinoma: a systematic review. *Br J Cancer* 2007; 96:1127–1134. 17406349 10.1038/sj.bjc.6603649 PMC2360117

[3] Liu F Li B Wei Y XRCC1 genetic polymorphism Arg399Gln and hepatocellular carcinoma risk: a meta-analysis. *Liver Int* 2011; 31:802–809. 21645210 10.1111/j.1478-3231.2011.02508.x

[4] Arzumanyan A Reis HM Feitelson MA Pathogenic mechanisms in HBV- and HCV-associated hepatocellular carcinoma. *Nat Rev Cancer* 2013; 13:123–135. 23344543 10.1038/nrc3449

[5] Scalera A Tarantino G Could metabolic syndrome lead to hepatocarcinoma via non-alcoholic fatty liver disease? *World J Gastroenterol* 2014; 20:9217–9228. 25071314 10.3748/wjg.v20.i28.9217 PMC4110551

[6] Bowen DG Walker CM Adaptive immune responses in acute and chronic hepatitis C virus infection. *Nature* 2005; 436:946–952. 16107834 10.1038/nature04079

[7] Smith TR Miller MS Lohman KK DNA damage and breast cancer risk. *Carcinogenesis* 2003; 24:883–889. 12771032 10.1093/carcin/bgg037

[8] Friedberg EC How nucleotide excision repair protects against cancer. *Nat Rev Cancer* 2001; 1:22–33. 11900249 10.1038/35094000

[9] Flejter WL McDaniel LD Johns D Correction of xeroderma pigmentosum complementation group D mutant cell phenotypes by chromosome and gene transfer: involvement of the human ERCC2 DNA repair gene. *Proc Natl Acad Sci USA* 1992; 89:261–265. 1729695 10.1073/pnas.89.1.261 PMC48216

[10] Coin F Marinoni JC Rodolfo C Mutations in the XPD helicase gene result in XP and TTD phenotypes, preventing interaction between XPD and the p44 subunit of TFIIH. *Nat Genet* 1998; 20:184–188. 9771713 10.1038/2491

[11] Pabalan N Francisco-Pabalan O Sung L Meta-analysis of two ERCC2 (XPD) polymorphisms, Asp312Asn and Lys751Gln, in breast cancer. *Breast Cancer Res Treat* 2010; 124:531–541. 20379847 10.1007/s10549-010-0863-6

[12] Lunn RM Helzlsouer KJ Parshad R XPD polymorphisms: effects on DNA repair proficiency. *Carcinogenesis* 2000; 21:551–555. 10753184 10.1093/carcin/21.4.551

[13] Seker H Butkiewicz D Bowman ED Functional significance of XPD polymorphic variants: attenuated apoptosis in human lymphoblastoid cells with the XPD 312 Asp/Asp genotype. *Cancer Res* 2001; 61:7430–7434. 11606376

[14] Qiao Y Spitz MR Shen H Modulation of repair of ultraviolet damage in the host-cell reactivation assay by polymorphic XPC and XPD/ERCC2 genotypes. *Carcinogenesis* 2002; 23:295–299. 11872635 10.1093/carcin/23.2.295

[15] Zhang RC Mou SH Polymorphisms of excision repair gene XPD Lys751Gln and hOGG1 Ser326Cys might not be associated with hepatocellular carcinoma risk: a meta-analysis. *Tumour Biol* 2013; 34:901–907. 23271362 10.1007/s13277-012-0625-7

[16] Wu JS Chen YP Wang LC Implication of polymorphisms in DNA repair genes with an increased risk of hepatocellular carcinoma. *Genet Mol Res* 2014; 13:3812–3818. 24938468 10.4238/2014.May.16.5

[17] Yao JG Huang XY Long XD Interaction of DNA repair gene polymorphisms and aflatoxin B1 in the risk of hepatocellular carcinoma. *Int J Clin Exp Pathol* 2014; 7:6231–6244. 25337275 PMC4203246

[18] Guo LY Jin XP Niu W Association of XPD and XRCC1 genetic polymorphisms with hepatocellular carcinoma risk. *Asian Pac J Cancer Prev* 2012; 13:4423–4426. 23167354 10.7314/apjcp.2012.13.9.4423

[19] Zeng XY Qiu XQ Ji L Study on the relationship between hepatocellular carcinoma and the interaction between polymorphisms in DNA repair gene XPD and environmental factors. *Zhonghua Liu Xing Bing Xue Za Zhi* 2009; 30:702–705. 19957595

[20] Jiang Y Yin MW Yu Z Relationship of hOGG1 and XPD gene polymorphisms with the risk of gastric cancer, liver cancer, and colorectal cancer. *Zhong Guo Zhong Liu Lin Chuang* 2012; 39:1358–1362.

[21] Stroup DF Berlin JA Morton SC Meta-analysis of observational studies in epidemiology: a proposal for reporting. Meta-analysis of Observational Studies in Epidemiology (MOOSE) group. *JAMA* 2000; 283:2008–2012. 10789670 10.1001/jama.283.15.2008

[22] Higgins JP Thompson SG Quantifying heterogeneity in a meta-analysis. *Stat Med* 2002; 21:1539–1558. 12111919 10.1002/sim.1186

[23] DerSimonian R Laird N Meta-analysis in clinical trials. *Control Clin Trials* 1986; 7:177–188. 3802833 10.1016/0197-2456(86)90046-2

[24] Mantel N Haenszel W Statistical aspects of the analysis of data from retrospective studies of disease. *J Natl Cancer Inst* 1959; 22:719–748. 13655060

[25] Duval S Tweedie R Trim and fill: a simple funnel-plot-based method of testing and adjusting for publication bias in meta-analysis. *Biometrics* 2000; 56:455–463. 10877304 10.1111/j.0006-341x.2000.00455.x

[26] Yue AM Xie ZB Guo SP Implication of polymorphisms in DNA repair genes in prognosis of hepatocellular carcinoma. *Asian Pac J Cancer Prev* 2013; 14:355–358. 23534753 10.7314/apjcp.2013.14.1.355

[27] Ye X Peng T Liu T Association between aldehyde dehydrogenase-2/cytochrome P450 2E1 genetic polymorphism and habit of alcohol drinking and the susceptibility of hepatocellular carcinoma. *Wei Sheng Yan Jiu* 2010; 39:42–45. 20364586

[28] Gulnaz A Sayyed AH Amin F Association of XRCC1, XRCC3, and XPD genetic polymorphism with an increased risk of hepatocellular carcinoma because of the hepatitis B and C virus. *Eur J Gastroenterol Hepatol* 2013; 25:166–179. 23044807 10.1097/MEG.0b013e328359a775

[29] Yuan T Deng S Liu H Relationship between XRCC1 and XPD polymorphisms and the risk of the development of hepatocellular carcinoma: a case–control study. *Exp Ther Med* 2012; 4:285–290. 22970032 10.3892/etm.2012.581 PMC3439166

[30] Chen CC Yang SY Liu CJ Association of cytokine and DNA repair gene polymorphisms with hepatitis B-related hepatocellular carcinoma. *Int J Epidemiol* 2005; 34:1310–1318. 16172101 10.1093/ije/dyi191

[31] Long XD Ma Y Zhou YF XPD codon 312 and 751 polymorphisms, and AFB1 exposure, and hepatocellular carcinoma risk. *BMC Cancer* 2009; 9:400. 19919686 10.1186/1471-2407-9-400 PMC2781019

[32] Xu L Wu Y Jing Y A case–control study on polymorphism of DNA repair gene XPD and susceptibility to hepatocellular carcinoma. *Tumor* 2004; 24:526–530.

[33] Cui X Su H A case–control study on the polymorphism of gene XPD and the susceptibility of primary hepatic carcinoma. *Med J Chin People Health* 2010; 22:912–916.

[34] Xie W Association between the nucleotide excision repair (NER) genes polymorphisms and genetic susceptibility and clinical phenotype of hepatocellular carcinoma. 2007.

[35] Duell EJ Wiencke JK Cheng TJ Polymorphisms in the DNA repair genes XRCC1 and ERCC2 and biomarkers of DNA damage in human blood mononuclear cells. *Carcinogenesis* 2000; 21:965–971. 10783319 10.1093/carcin/21.5.965

[36] Benhamou S Sarasin A ERCC2/XPD gene polymorphisms and lung cancer: a HuGE review. *Am J Epidemiol* 2005; 161:1–14. 15615908 10.1093/aje/kwi018

[37] Taylor EM Broughton BC Botta E Xeroderma pigmentosum and trichothiodystrophy are associated with different mutations in the XPD (ERCC2) repair/transcription gene. *Proc Natl Acad Sci USA* 1997; 94:8658–8663. 9238033 10.1073/pnas.94.16.8658 PMC23065

[38] Hou SM Falt S Angelini S The XPD variant alleles are associated with increased aromatic DNA adduct level and lung cancer risk. *Carcinogenesis* 2002; 23:599–603. 11960912 10.1093/carcin/23.4.599

[39] Akkiz H Bayram S Bekar A No association of pre-microRNA-146a rs2910164 polymorphism and risk of hepatocellular carcinoma development in Turkish population: a case–control study. *Gene* 2011; 486:104–109. 21807077 10.1016/j.gene.2011.07.006

[40] Zhang Z Wang M Wu D P53 codon 72 polymorphism contributes to breast cancer risk: a meta-analysis based on 39 case–control studies. *Breast Cancer Res Treat* 2010; 120:509–517. 19629678 10.1007/s10549-009-0480-4

[41] Mitchell AA Cutler DJ Chakravarti A Undetected genotyping errors cause apparent overtransmission of common alleles in the transmission/disequilibrium test. *Am J Hum Genet* 2003; 72:598–610. 12587097 10.1086/368203 PMC1180236

[42] Ioannidis JP Patsopoulos NA Evangelou E Uncertainty in heterogeneity estimates in meta-analyses. *BMJ* 2007; 335:914–916. 17974687 10.1136/bmj.39343.408449.80 PMC2048840

